# Phenotype of insulin-dependent diabetes in chronic undernutrition: beta cell stress and immune dysfunction—a rural sub-Saharan perspective on type 5 diabetes

**DOI:** 10.1007/s00125-025-06553-w

**Published:** 2025-09-29

**Authors:** Elisabeth R. Trimble, David I. W. Phillips, Shitaye A. Balcha

**Affiliations:** 1 Centre for Public Health, Institute for Clinical Science, Queen’s University Belfast, Belfast, UK; 2 MRC Lifecourse Epidemiology Centre, University of Southampton, Southampton General Hospital, Southampton, UK; 3 Department of Internal Medicine, Gondar University Hospital, Gondar, Ethiopia

**Keywords:** Emigration, Genetics, Parasitic infection, Review, Rural, Sub-Saharan Africa, Type 1 diabetes, Type 5 diabetes, Undernutrition

## Abstract

In economically developed countries most lean individuals presenting with insulin-dependent diabetes have autoimmune type 1 diabetes. However, in many rural areas of low- and middle-income countries (LMIC), 40–50% of individuals with a similar clinical presentation are negative for diabetes-associated autoantibodies at initial clinical presentation. The phenotype differs from the classical presentation of type 1 diabetes even in those with evidence of an autoimmune process: altered autoantibody profile; later peak age of onset; and, in those with post-pubertal clinical presentation, more marked male predominance. The incidence of insulin-dependent diabetes in LMIC is low, even when assessing those with and without autoantibodies together. A framework of possible pathophysiological mechanisms underlying the observed phenotypic differences is presented to explain how chronic undernutrition and micronutrient deficiencies might alter the presentation of insulin-dependent diabetes. Inhabitants of rural sub-Saharan Africa (SSA) depend almost entirely on staple foods grown locally in nutrient-deficient soil. The resulting chronic undernutrition, often intergenerational, affects linear growth and body morphology, and has direct immune and non-immune effects on beta cell development and function. Undernutrition directly affects thymic function, alters the autoimmune profile and is often associated with social deprivation and parasitic infection, both of which can delay and modify the (auto)immune response. Non-immune effects of undernutrition include beta cell stress, associated with apoptosis and formation of neoantigens. That environmental effects of undernutrition and social deprivation affect the altered insulin-dependent diabetes phenotype is shown by the movement back towards a classical type 1 diabetes phenotype in offspring of emigrants from SSA who are born in and develop insulin-dependent diabetes in an economically developed country. The degree of phenotype change depends on how long the parents have lived in their adopted country. It has recently been proposed that insulin-dependent diabetes in those who are mal/undernourished be called type 5 diabetes. There is need for clinician recognition of the altered phenotype(s) of insulin-dependent diabetes resulting from chronic undernutrition in rural LMIC. Additionally, changes in agricultural practice are needed to improve the nutrient content of food consumed by the rural population.

**Graphical Abstract:**

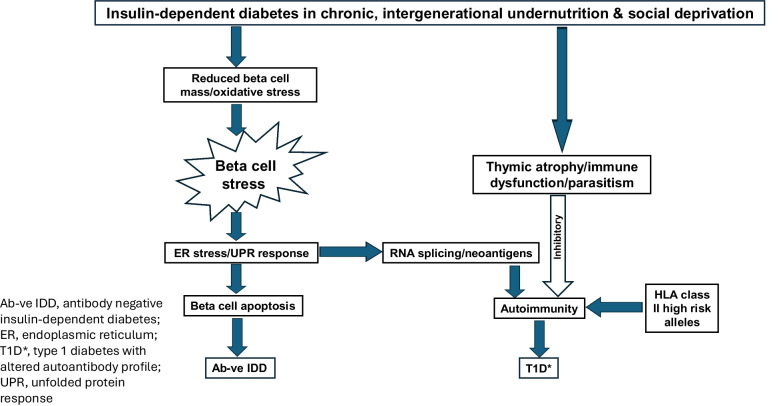

**Supplementary Information:**

The online version contains a slide of the figure for download, which is available to authorised users at 10.1007/s00125-025-06553-w.

## The unusual phenotype of insulin-dependent diabetes in sub-Saharan Africa

The phenotype of insulin-dependent diabetes has been well characterised in economically developed countries where autoimmune type 1 diabetes accounts for the overwhelming majority of all insulin-dependent diabetes. However, insulin-dependent diabetes (with or without autoimmune input) has been largely neglected in low- and middle- income countries (LMIC), although these countries represent 86% of the world’s population. Published evidence suggests that people in sub-Saharan Africa (SSA) presenting with clinical features of type 1 diabetes have a phenotype differing from that of type 1 diabetes in the economically developed world. In their recent publication on type 1 diabetes phenotype in SSA, Katte et al reviewed in detail publications from a variety of countries, representing widely different regions of SSA [1]. Most of the people in these regions are composed of indigenous African ethnic groups. Some countries, such as Eritrea, Sudan and Mali, have an Arab admixture while others have had influences from the Indian subcontinent. In their review, there is a general consensus on the phenotype of type 1 diabetes with respect to markers of autoimmunity, peak age of onset and degree of male predominance in adult-onset diabetes but exceptionally few published data on the detailed prevalence of HLA class II alleles. There are limited data on the occurrence of autoantibodies in newly presenting individuals, necessary as their prevalence changes with time from first clinical presentation. Available data, however, do suggest that newly presenting individuals have an autoantibody profile differing from that found in the economically developed world in that, while many have anti-GAD antibodies, relatively few have anti-IA-2 and anti-ZnT8 antibodies [1]. By contrast, at clinical onset, most individuals with type 1 diabetes who are of European background have multiple autoantibodies [2]. Next, the peak age of clinical onset in SSA is in the mid-20s, a decade later than in economically developed countries [3–6]; after this early peak, a percentage of individuals present in later life in both LMIC [3] and European countries [7, 8]. Finally, although there is no consistent sex difference in incident cases among pre-pubertal children, onset in post-pubertal young adults exhibits significant male predominance both in LMIC [9, 10] and in economically developed countries [7, 8, 11, 12], with male predominance being much greater in socially deprived rural communities of SSA [6]. These sex differences do not appear to be related to islet cell autoimmunity [12]. In addition to phenotypic differences, the incidence of type 1 diabetes is lower in SSA than in economically developed countries [6], in line with the well-known geoepidemiological gradient of all autoimmune diseases that are lower in LMIC than in economically developed countries [13]. However, specifically in type 1 diabetes, death before getting access to insulin probably also contributes to the low recorded incidence. One published exception is Eritrea where the incidence of childhood-onset type 1 diabetes is moderately high [10]. These presentational differences in a disease that symptomatically resembles type 1 diabetes at its clinical onset raise questions as to the aetiology and pathogenesis of the disease in these mainly thin, Black African communities and whether they result from differences in the prevalence of high-risk HLA genes, environmental variation or their interaction.


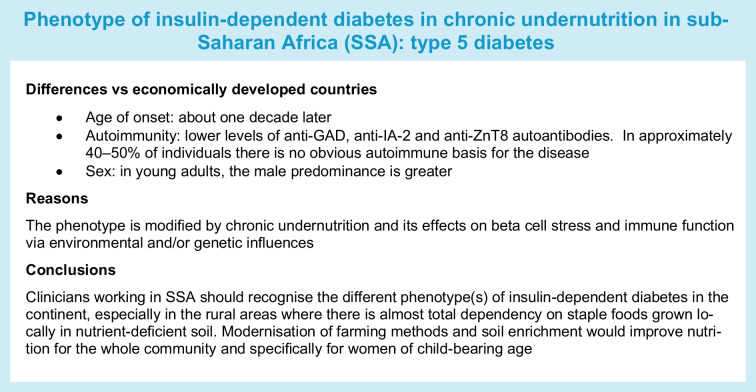


## The role of genetics

Tishkoff and colleagues have documented the vast genetic diversity of SSA, believed to be much greater than among non-Africans [14, 15]. Some genetic loci show signatures of adaptation to different environments, diets and pathogens [15], and are probably combined with more recent epigenetic changes. Additionally, in some northern areas close to ancient European and Arab trade routes, genetic elements from these trading groups have been incorporated into African genomes over many centuries [15]. For example, the Amhara of North-West Ethiopia, who have formed the basis of our type 1 diabetes studies in Africa, display some features associated with people of European descent; they have a distinct genome that, of those African genomes studied in detail, exhibits fewest differences from non-African groups [15]. It has been shown that type 1 diabetes in this group is associated with HLA class II *DR3* and *DR4* [16] as is the case for those of European background. However, elsewhere in Africa the situation may be different, with differences between West African (Cameroon) and European (Belgium) individuals [17]. With more in-depth knowledge of the diverse African genomes, it will be interesting to see if or how they alter the phenotype of type 1 diabetes.

There is, however, persuasive evidence that the African type 1 diabetes phenotype is modified or even disappears following migration to an economically developed country, suggesting the operation of environmental rather than genetic factors in the altered phenotype. When large numbers of Ethiopian Jews migrated from the rural Amhara region of North-West Ethiopia to Israel, there was a slow decrease in the age of clinical onset of type 1 diabetes (in those with at least two HLA class II, high-risk alleles), in relation to the time their Ethiopian-born parents were resident in Israel [18]. At the same time, the childhood incidence of type 1 diabetes, which had been extremely low in Ethiopia (2.75/100,000) [9], rose to be one of the highest among the Jewish communities in Israel (18.2/100,000), second only to that of Yemenite Jews [19]. A similar effect on the incidence of type 1 diabetes was reported among the Swedish-born children of East African migrants. The offspring of mothers born in East Africa but living in Sweden for 11 years or more had a 22% higher incidence of type 1 diabetes than those with mothers living in Sweden for 5 years [20]; the risk of type 1 diabetes increased with the mothers’ duration of stay in this economically developed country, underscoring the influence of environment on the incidence of type 1 diabetes.

## Putative environmental factors

Evidence from twin concordance studies [21] and epidemiological observations have implicated possible environmental factors in the development of type 1 diabetes. A wide range of environmental factors has been investigated, including nutrition, viral infections and gut microbiota. Several other factors have received variable support (see Stene et al for an extensive review [22]). However, most of these studies have been carried out in the economically developed world in the context of overnutrition and obesity. Consequently little is known as to whether these factors are relevant in the resource-poor world. The effects of nutrition in diabetes have most commonly been studied in relation to overnutrition and obesity-related type 2 diabetes, reviewed in [23]; additionally, a large body of data from human and animal studies has shown that adverse early environments interact with postnatal factors (excess weight gain/obesity, smoking, poor diets and physical inactivity) to increase the risk of type 2 diabetes [24]. In some large urban areas where there is access to western-style energy-dense foods, the ‘double burden’ of disease occurs [25], that is, obesity and insulin resistance co-existing with a continuing state of malnutrition. Since much of the diabetes research focus in LMIC has been on the rapid rise of conventional, obesity-related type 2 diabetes in large urban centres, less attention has been paid to lean, insulin-dependent diabetes, which is one of the commoner forms of diabetes in the poorer rural areas where undernutrition is more or less continuous and the lifestyle is active [9].

## Undernutrition and stunting in SSA

Overall, about 60% of the 1.54 billion population of SSA comprises rural dwellers, although this varies from country to country. For example in Ethiopia, with the second-largest population in SSA (126.5 million), 78% of the population lives in rural areas (as of 2023), while in Nigeria, with the largest population in SSA (224 million in 2023), the rural and urban populations are almost the same. These rural communities are often poor and depend on unproductive, subsistence agriculture for their livelihood. Due to repeated crop failures and outdated farming methods, which have depleted the soil of important nutrients, locally grown staple foods and traditional diets are often nutrient deficient. Food deficits are frequent and there is vulnerability to flooding, drought and pests. As a result, protein-energy deficiency and micronutrient deficiency are common. Urban areas are less affected as they are not totally dependent on locally grown foods and have greater dietary diversity [26, 27]. In the rural communities, chronic undernutrition is frequently associated with other forms of deprivation, such as lack of access to clean water and sanitation, conditions where infestation with parasites is almost universally present and which may exacerbate nutritional deficiencies [28].

As a result, many mothers in SSA are underweight, with BMIs <18.5 kg/m^2^, and have related micronutrient deficiencies including zinc, copper, iron, selenium, iodine, vitamins A, D and B_12_, and folic acid. Rural women of child-bearing age and lactating mothers are particularly affected, and more so if the mother is poor and uneducated [29–32]. In these conditions of chronic intergenerational undernutrition the genomes of both father [33] and mother [34] will have adapted to the prevalent nutritional conditions and their altered (epi)genomes will input to the fetal genome. One important but poorly understood issue is the emerging evidence that changes in parental genomes have a greater effect on the metabolism of male offspring compared with female offspring [35], and this difference is greater in poor rural communities [36]. The effects on child growth and development and immunity [37] in this context are complex and will depend on the degree and chronicity of under/malnutrition, whether undernutrition is intergenerational (with epigenome changes in the parents), the timing of undernutrition and on the elements in which the diet is deficient. Nevertheless, nutritional stunting is widespread throughout SSA, affecting 36.6% of children under the age of 5 years [36]. Stunting is a non-invasive, easily accessible indicator of poor nutrition during the growth period and frequently leads to more detailed investigations, such as those which involve pancreatic and hepatic function.

## Undernutrition and insulin-dependent diabetes

There is a long history of moderate to severe malnutrition being associated with a condition resembling type 1 diabetes in different parts of the world. This form of diabetes, previously called malnutrition-related diabetes mellitus has also been associated with immune dysfunction and has shown some presentational differences among the different global regions, such as India [38], Jamaica [39] and SSA [40], and is still reported from many LMIC [41]. In Ethiopia, insulin-dependent diabetes is strongly linked with markers of poverty and nutritional stunting. Young adult men presenting with symptoms indistinguishable from type 1 diabetes were up to 2 cm shorter than their non-diabetic male control counterparts, and had evidence of skeletal disproportion [42]. Additionally, male individuals with insulin-dependent diabetes in rural areas were more stunted than those in urban areas [6], although the female sex displayed similar heights for those with versus without diabetes. These studies suggest a greater effect of undernutrition-related stunting in male individuals with symptoms of type 1 diabetes, especially rural males in a largely rural community [6]. This is in line with research which shows that metabolic traits modified by suboptimal early environments preferentially affect male individuals [35]. The IDF has suggested that insulin-dependent diabetes associated with under/malnutrition be called type 5 diabetes (without reference to whether it is autoimmune or not) and has set up a working group to establish appropriate diagnostic criteria (see idf.org/news/new-type-5-diabetes-working-group/, accessed 29 April 2025).

## Possible mechanisms linking undernutrition with insulin-dependent diabetes

**Fig. 1:**
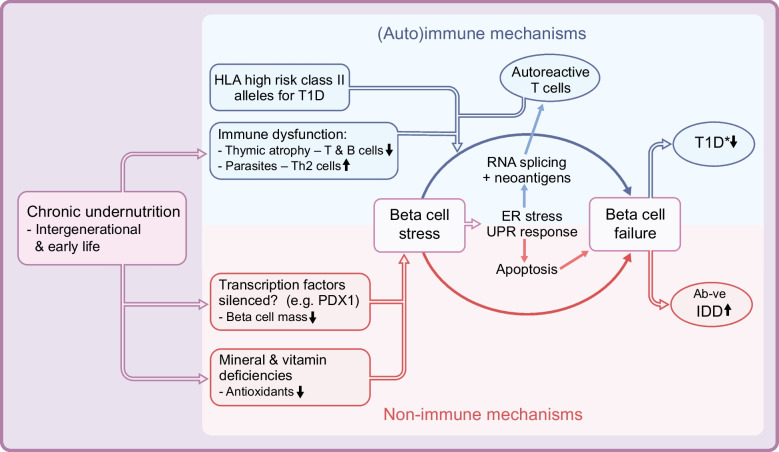
Mechanisms involved in beta cell stress and immune dysfunction in chronic intergenerational undernutrition, showing effects on type 1 diabetes with altered autoantibody profile (T1D*) and autoantibody-negative insulin-dependent diabetes (Ab-ve IDD). **Beta cell stress**: chronic intergenerational undernutrition probably causes epigenetic silencing of growth and development transcription factors, thus limiting beta cell mass. It is also associated with mineral and vitamin deficiencies that reduce antioxidant levels. Both reduced beta cell mass and lower antioxidant levels are associated with beta cell stress. **Immune dysfunction**: undernutrition in utero causes thymic atrophy with reduction in both T and B cell lineages. In rural areas, with poor sanitation, parasitic infection is widespread. The resulting Th2 immune response (e.g. to helminth infection) opposes the Th1 cell response active in the development of type 1 diabetes, and has an overall negative effect on autoimmune responses. **ER**: as beta cell stress increases, the ER is a key site that, due to oxidative stress, may not be able to fold all the proinsulin molecules arriving in it. This activates the UPR, and as stress continues to rise there are different outcomes. On the one hand, apoptosis occurs, which can lead to increased production of Ab-ve IDD. On the other hand, RNA splicing and neoantigen formation occurs from proinsulin fragments and post-translationally altered molecules; these neoantigens are not native molecules, are not represented in the thymus and are not recognised as ‘self’. The neoantigens react with autoreactive T cells. As apoptosis increases and more beta cells die, Ab-ve IDD increases as a proportion of total insulin-dependent diabetes. The degree of autoimmune type 1 diabetes developing in those with high-risk HLA class II alleles will depend on several variables, including the degree to which the thymus has been affected by undernutrition, the reaction of autoreactive T cells to neoantigens and the degree by which these factors are opposed by parasitic infections that stimulate a Th2 response. Type 1 diabetes will have an altered phenotype (T1D*), its proportion of total insulin-dependent diabetes decreasing as Ab-ve IDD increases. This figure is available as a downloadable slide

There is increasing evidence that undernutrition at various stages of life can affect beta cell development and beta cell function. These alterations result from both (auto)immune and non-immune mechanisms that combine to modify the phenotype of type 1 diabetes and increase the development of antibody-negative insulin-dependent diabetes (summarised in Fig. 1). In animals exposed to early-life undernutrition there is epigenetic silencing of transcription factors important for hepatic and pancreatic development (e.g. pancreatic and duodenal homeobox 1 [PDX1], insulin-like growth factor 2 [IGF2] and hepatocyte nuclear factor 4α [HNF4α]) [43–45]. These changes have been linked to very restricted replication of beta cells in utero and to reduced beta cell mass in rodents. It is of interest that mutations in some of the same transcription factors (e.g. HNF4α and PDX1) cause MODY [46]. If, in humans, these transcription factors (and others) were to undergo epigenetic silencing in utero (not yet proven)*,* beta cell replication would be reduced. What is known is that, unlike rodents, humans have very restricted beta cell replication after 5 years of age [47, 48], except for limited replication in obesity and pregnancy. Relevant reports on pancreas size in humans are extremely rare, and show that with or without evidence of autoimmunity, non-diabetic first-degree relatives of individuals with type 1 diabetes have smaller pancreases than those of the general population [49]; the reason for this is obscure.

In healthy individuals, glucose metabolism depends on the regulation of synthesis–secretion coupling in the beta cell, which has to respond immediately to changes in blood glucose levels with an up to 50-fold increase in insulin synthesis. These responses are highly dependent on rapid increases in oxidative processes in the mitochondria and the endoplasmic reticulum (ER). The production of energy (ATP) in the mitochondria is a multi-step oxidative process that also produces significant amounts of reactive oxygen species (ROS), with pathways for removal in health [50]. Even in the healthy state, the beta cell is deficient in antioxidants compared with other pancreatic cells, especially catalase and glutathione peroxidase [51]. Although it can normally undertake mitophagy in health, the situation is different in undernutrition with a restricted diet, and where antioxidant levels are even lower. In these conditions, as the amount of oxidative stress in the mitochondria augments, more mitochondria are damaged, the pathways for mitophagy become overloaded and the damaged mitochondria and beta cells become dysfunctional [50]. Another major stress occurs when the ER is suddenly flooded with newly synthesised proinsulin, which requires folding with the insertion of three disulphide bonds. Producing these structural changes in proinsulin leaves the ER in a constant state of oxidative stress because proper folding requires oxidative processes [52]. The excess unfolded protein has to be dealt with by a process known as the unfolded protein response (UPR), which has two modes depending on the level of stress. At an early stage of the UPR, an important aim is to lower the level of mRNA translation and reduce the burden on the ER (adaptive stage). However, if stress is prolonged or made worse by environmental triggers, such as a low-energy diet with reduced antioxidant capacity, then the next stage of the UPR (terminal stage) is activated. This leads to apoptosis, as reviewed by Piganelli et al [53]. In circumstances of undernutrition, apoptosis is probably an early step in the development of lean type 2 diabetes (which is not the subject of this paper) but when undernutrition is more severe, it may lead to autoantibody-negative insulin-dependent diabetes (Fig. 1).

These abnormal beta cell processes are also involved in the production of post-translationally modified neoantigens. Even in healthy individuals, the beta cell may not complete the folding of all the proinsulin supplied to the ER, suggesting that the cell is frequently working at maximum capacity [54]. This results not only in apoptosis but also in the production of fragments of proinsulin or post-translationally transformed molecules, which act as neoantigens that are not recognised as ‘self’ and thus evade immune tolerance. These fragments/molecules are detected by autoreactive T cells specific for these modified antigens that circulate in individuals with type 1 diabetes [55]. It is important to note that autoreactive T cells circulate at low levels even in health [56]. However, most people with low levels of autoreactive T cells do not develop diabetes and it may take a trigger, such as a viral infection or a diet that increases beta cell stress (as in prolonged undernutrition), to induce the autoimmune process in those with high-risk HLA alleles for type 1 diabetes. Unfortunately it is not possible, as yet, to test routinely for any putative autoantibodies to the neoantigens nor to quantify the role of increased levels of neoantigens, as putative triggers, in the autoimmune response. For recent detailed reviews of the cellular mechanisms by which the beta cell contributes to its own death, see Piganelli et al [53] and Roep et al [57]; this idea was first mooted by Bottazzo 40 years ago [58].

## Undernutrition and immunity

Immunity is altered by undernutrition in utero; this results in thymic disorganisation and atrophy, with thymocyte depletion and some loss of the usual cortico-medullary architecture of the thymus [59]. In addition to effects of undernutrition on general immunity [37, 60], there are implications for T cell selection, immune tolerance and long-term effects on immunity [61]. Continued undernutrition in postnatal life negatively affects T cell bioenergetics, metabolism and function [62]. Additionally, there are many infective agents with more specific effects on autoimmune responses in type 1 diabetes and other autoimmune diseases [63]; these include parasites. For the undernourished living with poor sanitation in rural areas of SSA, parasitic infection is common. As an example, in helminth infections, which are widespread, T cells are stimulated to give a major T helper 2 (Th2) response, thus opposing the T helper 1 (Th1) effects associated with autoimmune cell death in type 1 diabetes (Fig. 1). Helminths have been shown to delay the onset or inhibit the development of type 1 diabetes [64, 65]. Parasitic infections were probably more widespread historically when suboptimal sanitation was the norm and were possibly influential in curtailing the global incidence of autoimmune diseases [63]. However, with better sanitation and fewer parasitic infections in the economically developed world, autoimmune diseases have increased and contribute to the present geoepidemiological gradient for autoimmune disease [13, 66].

## Insulin-dependent diabetes in rural SSA: autoantibody positive and autoantibody negative

First, the expected consequence of an increase in non-immune-related apoptosis is a higher proportion of autoantibody-negative insulin-dependent diabetes. Second, islet autoimmunity will be modified, especially in those with high-risk HLA class II susceptibility alleles, due to the combined effects of undernutrition on thymic function, the production of post-translationally modified neoantigens, and the effects of parasitism (see above). Due to widespread parasitic infection, autoimmune processes are also likely to be (time-)delayed [64, 65]. A recent study was undertaken in the Amhara of rural North-West Ethiopia who suffer from chronic intergenerational undernutrition and presumed thymic dysfunction. Consecutive, unselected individuals who presented clinically with symptoms indistinguishable from autoimmune type 1 diabetes had low C-peptide levels and were insulin-dependent from their initial presentation [16]. Of these individuals, 60.6% were autoantibody positive, most had anti-GAD autoantibodies, very few had autoantibodies to IA-2 or ZnT8, and 39.4% were autoantibody negative; the autoantibody-positive group were positive for *HLA-DRB3* and *HLA-DR4*. Although there was evidence of altered B cell function (autoantibodies), the level of T cell activity was not known. By contrast, in those with a European background a higher percentage are positive for autoantibodies at initial presentation [2]. A study from Vellore (India) also demonstrated heterogeneity with respect to the autoimmune basis of insulin-dependent diabetes in young adults [67]; the social circumstances of this Indian group were not dissimilar from those of rural Ethiopia. Autoantibody-negative insulin-dependent diabetes also occurs in economically developed countries with good nutrition; however, the percentage of total insulin-dependent diabetes is much lower [68] than that in Ethiopia or India at similar ages of onset [2]. Since in health the beta cell is working at or near maximum capacity with respect to the ER and proinsulin folding (see above) [54], there is almost certainly a low level of apoptosis in those who are not undernourished; this could contribute to the low level of autoantibody-negative insulin-dependent diabetes found in economically developed countries [2].

In conclusion, we postulate that both autoimmune type 1 diabetes and autoantibody-negative insulin-dependent diabetes are altered in conditions of intergenerational undernutrition and deprivation by a complex series of pathophysiological mechanisms. Chronic undernutrition in early life impairs beta cell development and, if continued, predisposes to beta cell stress. Stress occurs in the mitochondria and the ER and activates the UPR, which leads to apoptosis and autoantibody-negative insulin-dependent diabetes. Stress also leads to the production of proinsulin fragments and post-translationally modified proteins. These neoantigens, which are not seen as ‘self’, can escape immune tolerance. Thymus dysfunction due to in utero undernutrition is likely to modify the autoimmune response. In rural areas, autoimmune dysfunction is further altered by parasitism. However, the presence of a reduced diabetes-related autoantibody profile in 60% of individuals with insulin-dependent diabetes suggests that some autoimmunity remains. It is postulated that undernutrition in utero will cause thymic atrophy and alter T cell function but it is difficult to quantify the degree of T cell dysfunction in vivo in humans. When emigration is associated with better nutrition, access to clean water and better sanitation there is a gradual change in the phenotype in those with high-risk HLA class II alleles for type 1 diabetes towards a more classical presentation of the disease. In the disadvantaged areas of SSA there is a need for clinician recognition of the alterations to the phenotype(s) within insulin-dependent diabetes. Changes are also needed in the management of soil and soil enrichment in order to improve the mineral and micronutrient content of locally grown foods and, thus, reduce undernutrition.

## Supplementary Information

Below is the link to the electronic supplementary material.

Figure slide (PPTX 200 KB)

## Abbreviations

ER: Endoplasmic reticulum
HNF4α: Hepatocyte nuclear factor 4α
IGF2: Insulin-like growth factor 2
LMIC: Low- and middle-income countries
PDX1: Pancreatic and duodenal homeobox 1
SSA: Sub-Saharan Africa
Th1: T helper 1
Th2: T helper 2
UPR: Unfolded protein response
