# Supplementary material for: Phenotype of insulin-dependent diabetes in chronic undernutrition: beta cell stress and immune dysfunction—a rural sub-Saharan perspective on type 5 diabetes
Source: Diabetologia. 2025 Sep 29;68(12):2643–51. doi: 10.1007/s00125-025-06553-w (PMC12594637; doi:10.1007/s00125-025-06553-w)
Supplement: Supplementary file 1 — Figure slide (PPTX 200 KB) [file 125_2025_6553_MOESM1_ESM.pptx]

## Slide 1
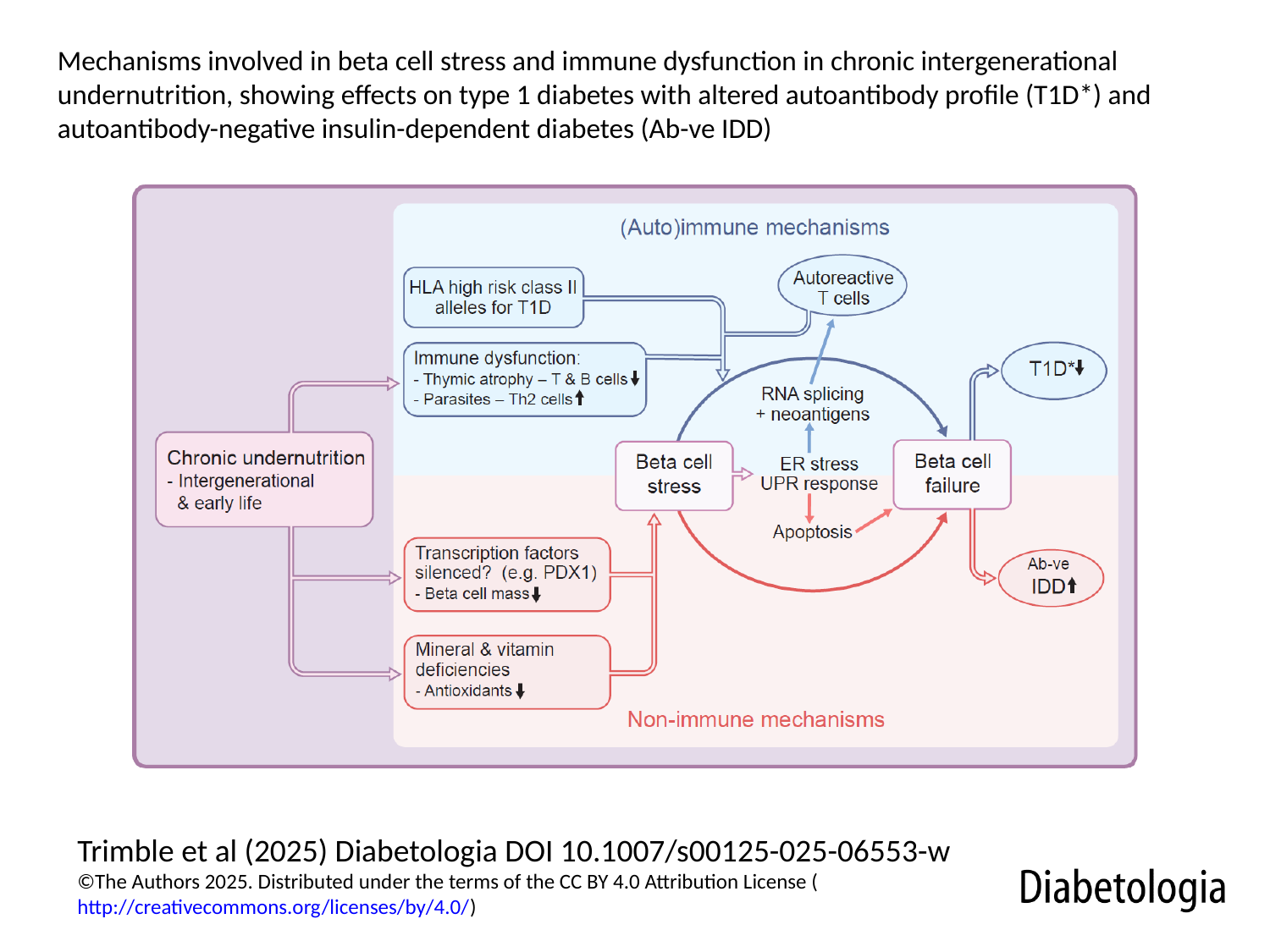

Mechanisms involved in beta cell stress and immune dysfunction in chronic intergenerational undernutrition, showing effects on type 1 diabetes with altered autoantibody profile (T1D*) and autoantibody-negative insulin-dependent diabetes (Ab-ve IDD)
Trimble et al (2025) Diabetologia DOI 10.1007/s00125-025-06553-w
©The Authors 2025. Distributed under the terms of the CC BY 4.0 Attribution License (http://creativecommons.org/licenses/by/4.0/)
